# Compact and Highly Sensitive NO_2_ Photoacoustic Sensor for Environmental Monitoring

**DOI:** 10.3390/molecules25051201

**Published:** 2020-03-07

**Authors:** Yufeng Pan, Lei Dong, Xukun Yin, Hongpeng Wu

**Affiliations:** 1 State Key Laboratory of Quantum Optics and Quantum Optics Devices, Institute of Laser Spectroscopy, Shanxi University, Taiyuan 030006, China; panyufeng2008@163.com (Y.P.); yinxkvip@163.com (X.Y.); wuhp@sxu.edu.cn (H.W.); 2 Collaborative Innovation Center of Extreme Optics, Shanxi University, Taiyuan 030006, China

**Keywords:** nitrogen dioxide sensor, photoacoustic spectroscopy, low-cost high power laser diode, compact low-noise photoacoustic detection module, outdoor environmental monitoring

## Abstract

A nitrogen dioxide (NO_2_) photoacoustic sensor for environmental monitoring was developed using a low-cost high-power laser diode emitting at 450 nm. A compact low-noise photoacoustic detection module was designed to reduce the sensor size and to suppress noise. A LabVIEW-based control system was employed for the sensor. The parameters of the sensor were studied in detail in terms of laser power and operating pressure. The linearity of the sensor response with laser power and NO_2_ concentration confirms that saturation does not occur. At atmospheric pressure, a 3*σ* detection limit of 250 ppt (part per trillion by volume) was achieved with a 1-s averaging time, which corresponds to the specific detectivity of 3.173 × 10^−9^ W cm^−1^ Hz^−1/2^. A 72 h outdoor continuous on-line monitoring of environmental NO_2_ was implemented to demonstrate the reliability and validity of the developed NO_2_ sensor.

## 1. Introduction

Nitrogen dioxide (NO_2_) is one of the primary atmospheric pollutants which mainly originates from natural lightning, vehicle exhaust emissions, and industrial combustion processes. It can cause the formation of acid rain and photochemical smog [1,2]. In 2016, Wang et al. verified that NO_2_ also represents one of the key contributors to haze [3]. Furthermore, NO_2_ even at low concentration levels is harmful to human health, especially to the respiratory tract. Therefore, the concentration level of NO_2_ is an important indicator in the daily air pollution index. The average mixing ratio of NO_2_ in the atmosphere is about 5–30 ppb but can be several orders of magnitude higher near its source and in heavily polluted areas [2,4]. The most widely used technique for the measurement of NO_2_ concentrations in environmental monitoring is the chemiluminescence technique. This method involves the reduction of NO_2_ to NO using heated (300–350 °C) molybdenum (Mo) surfaces or photolytic conversion followed by the gas-phase reaction between NO and O_3_ forming an electronically excited ${\mathrm{NO}}_{2}^{*}$ molecule that emits light, which is proportional to the NO concentration, and the NO_2_ concentration can be obtained from the NO concentration. However, when a chemiluminescence instrument with a molybdenum converter is used to measure environmental NO_2_ concentrations, the positive interferences caused by photochemically formed NOy species can appear, resulting in measured values significantly higher than the actual values. In contrast, the negative interferences caused by the photolysis of volatile organic compounds (VOCs) in the photolytic converter and consecutive peroxy radical reactions with NO can underestimate NO_2_ concentrations when a chemiluminescence instrument with a photolytic converter is used. The unreliable NO_2_ quantification can be obtained if these interferences are not taken into consideration in chemiluminescence techniques [5,6,7]. Therefore, in order to measure environmental NO_2_ concentrations accurately, it is of great significance to develop highly sensitive, interference-free, compact, and low-cost NO_2_ sensors.

Optical approaches offer the capability of interference-free direct measurements of NO_2_ concentrations without any sample preparation and chemical conversion [1,2,4,5,6,8]. Photoacoustic spectroscopy (PAS) is one of the most effective optical sensing techniques for trace gas detection due to its advantages of high sensitivity and selectivity as well as compact detection module size [8,9,10,11,12,13,14,15,16,17]. PAS-based sensors have been successfully applied in the field of environmental trace gas monitoring [15,16,17]. In the PAS technique, an acoustic wave is generated by the gas selective absorption of the modulated excitation light and converted into an electrical signal via an acoustic transducer (e.g., microphones, quartz tuning forks, or fiber tips); hence the target gas concentration can be obtained by detecting the PAS signal. A special feature of the PAS technique is that the photoacoustic detector is independent of the excitation optical wavelength, which means that any excitation light wavelength can be employed in the PAS technique. For NO_2_ detection, the NO_2_ fundamental vibrational transitions absorb strongly in the mid-infrared (MIR) region around 6.2 µm. In 2006, Pushkarsky et al. [18] reported a PAS-based sensor for NO_2_ detection at 6.25 µm by using a quantum cascade laser operating in an external grating cavity configuration. A 1*σ* detection limit of 0.5 ppb was achieved, which meets the needs of environmental NO_2_ detection. However, use of the quantum cascade laser made the sensor costly and complex. NO_2_ exhibits a strong absorption cross section with a broadband absorption feature in the green–blue–violet region, and a low-cost miniaturized blue laser diode (LD) is now commercially available. This provides the opportunity to develop the compact photoacoustic sensor for NO_2_ detection in environmental monitoring.

In this work, we develop a compact and highly sensitive NO_2_ photoacoustic sensor for environmental monitoring. The sensor employs a low-cost high-power LD emitting at 450 nm. A compact low-noise photoacoustic detection module was designed to detect the PAS signal. After parameters optimization and performance evaluation, a three-day outdoor continuous on-line monitoring of environmental NO_2_ concentrations was carried out using the reported NO_2_ sensor.

## 2. NO_2_ Sensor Design

### 2.1. Detection Wavelength and Excitation Light Source

**Figure 1:**
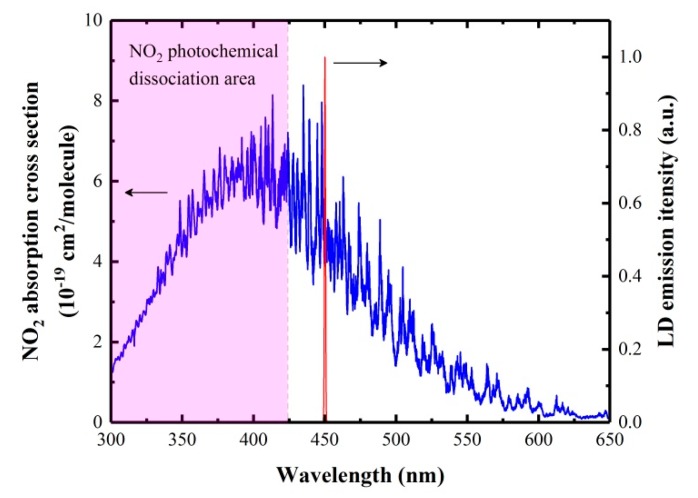
Nitrogen dioxide (NO_2_) absorption cross sections between 300–650 nm (blue) and the emission spectrum of the used laser diode (LD) (red).

**Figure 2:**
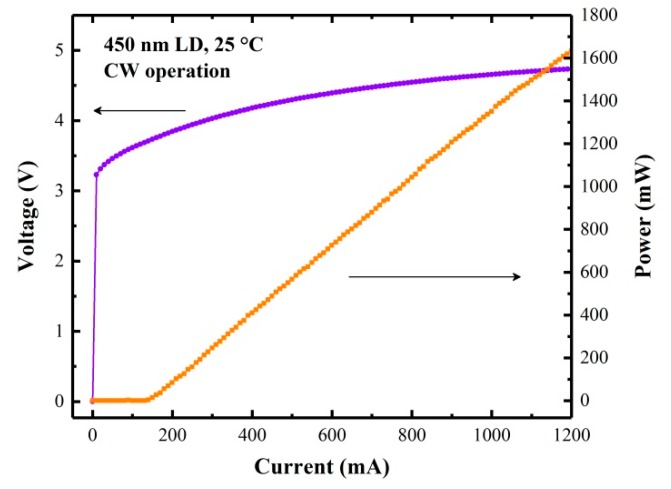
L-I-V (light-current-voltage) curve of the 450 nm LD.

According to the HITRAN database [19], NO_2_ exhibits a strong broadband absorption from 300 nm to 650 nm with a maximum cross section of 7.4 × 10^−19^ cm^2^/molecule at 414 nm. However, NO_2_ photochemical dissociation occurs below 424 nm, which is the main initial reaction of photochemical smog formation, as follows [1]:

(1) $$NO_{2}+hv\;\to NO+O$$

(2) $$O_{2}+O+M\to O_{3}+M$$

where M represents N_2_ or O_2_ or a third molecule. Moreover, a distinct advantage of PAS is that the performance of PAS-based sensors can be improved as the excitation light power is increased, since the photoacoustic signal scales linearly with excitation light power as long as saturation does not occur [20,21]. Hence, in order to avoid the NO_2_ photochemical dissociation and obtain photoacoustic signals as strong as possible, we selected a commercial high-power 450 nm LD (Thorlabs L450P1600MM) as an excitation light source for NO_2_ photoacoustic detection. It has a multi-transverse mode output with a beam divergence of *θ*_⊥_ ~23°, and *θ*_‖_ ~7°. Figure 1 shows the NO_2_ absorption cross sections between 300–650 nm and the emission spectrum of the used LD. The spectral linewidth of the LD is 0.8 nm. The measured L–I–V (light–current–voltage) curve at an operating temperature of 25 °C in continuous wave CW operation is shown in Figure 2. The maximum optical output power is 1.6 W at a forward voltage of 4.8 V when the LD was operated at a current of 1.2 A.

### 2.2. Photoacoustic Detection Module

**Figure 3:**
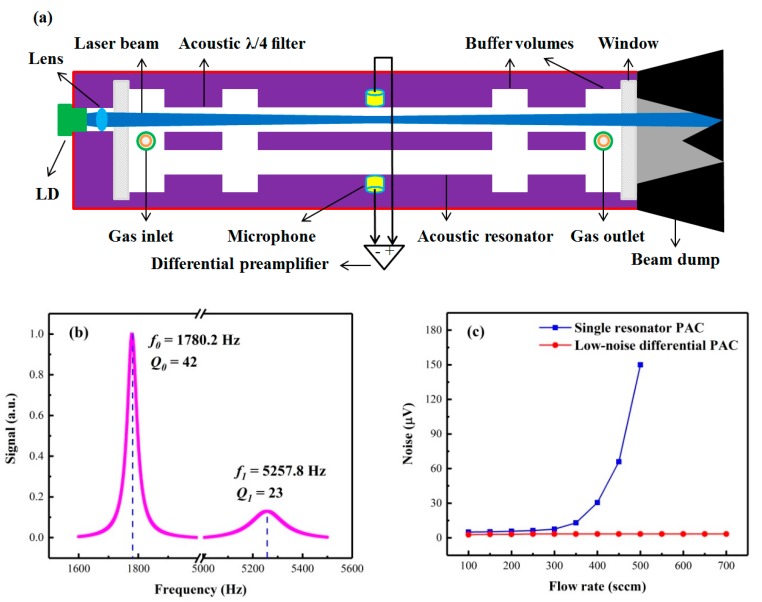
(**a**) Schematic of the photoacoustic detection module; (**b**) frequency response curves of the low-noise differential photoacoustic cell (PAC) displaying the fundamental and first longitudinal vibrational modes; (**c**) dependence of PAC noise on gas flow rate as measured on different PAC designs.

A photoacoustic detection module (PADM) consists of a LD, a collimating lens, a low-noise differential photoacoustic cell (PAC), a differential preamplifier, and a custom black-out rough beam dump as illustrated in Figure 3a. The LD was used as an excitation light source to excite the photoacoustic signal, and the LD beam was collimated to pass through the PAC by the collimating lens. The low-noise differential PAC was designed with a fully symmetrical geometry. It has two identical parallel stainless steel tube-shaped channels of 90 mm in length and 8 mm in diameter forming two acoustic resonators, which allows the collimated beam to pass through the photoacoustic cell easily. The acoustic resonators are placed between four buffer volumes with lengths of 10 mm and four acoustic *λ*/4 filters with inner diameters of 8 mm. The combination of buffer volumes and acoustic *λ*/4 filters efficiently reduces the noise originating from gas flow, windows, and external electromagnetic disturbances [15,22,23]. Two quartz windows with diameters of 25.4 mm were employed to insulate the photoacoustic cell from the surrounding environment, at the same time allowing access to the LD beam. The gas inlet and gas outlet holes were mounted in two symmetrical buffer volumes, respectively. When the laser intensity is modulated at the resonance frequency of the photoacoustic cell, a standing acoustic wave is generated by the optical absorption of the target gas, resulting in a maximum acoustic pressure in the middle of the acoustic resonator. Hence, two selected electret condenser cylindrical microphones with the same frequency response sensitivities were installed in the middle of each resonator and embedded into the walls to detect the acoustic signal. The photoacoustic signal is generated in only one acoustic resonator since the laser irradiates only one of them. All noise components that are coherent in both acoustic resonators can be eliminated by using a custom transimpedance differential preamplifier. The inner surface of the acoustic resonators, the buffer volumes, as well as the acoustic *λ*/4 filters, were polished in order to facilitate the formation of standing waves and further suppress the noise resulting from reflected or scattered light in the acoustic resonators. These designs improve the signal-to-noise ratio (SNR) of the sensor and reduce the interference of ambient noise greatly. After passing through the resonators, the high power laser light was collected and absorbed by the beam dump. The LD, the collimating lens, and the beam dump were mounted inside the photoacoustic cell; this makes the PADM compact and robust, which optimally matches the high-power laser beam.

Figure 3b shows the normalized frequency response curves of the low-noise differential PAC, displaying the fundamental and first longitudinal vibrational modes of the PAC at resonance frequencies of *f*_0_ = 1780.2 Hz and *f*_1_ = 5257.8 Hz, with quality factors of *Q*_0_ = 42 and *Q*_1_ = 23, respectively. Comparing both signal amplitudes, the fundamental longitudinal vibrational mode was chosen for the following experiments. The noise of the PAC as a function of the gas flow rate is depicted in Figure 3c. The results show that the noise remained minimal when the flow rate varied from 100 sccm (standard-state cubic centimeter per minute) to 700 sccm. For comparison, the noise generated by a single resonator PAC with identical geometrical dimensions as the designed PAC, but without the differential configuration and the acoustic *λ*/4 filters, is also shown in Figure 3c. When the gas flow rate exceeded 300 sccm, an obvious noise increase was observed. This result means the designed low-noise differential PAC can effectively suppress the gas flow noise when a large flow rate is used.

### 2.3. NO_2_ PAS Sensor System

**Figure 4:**
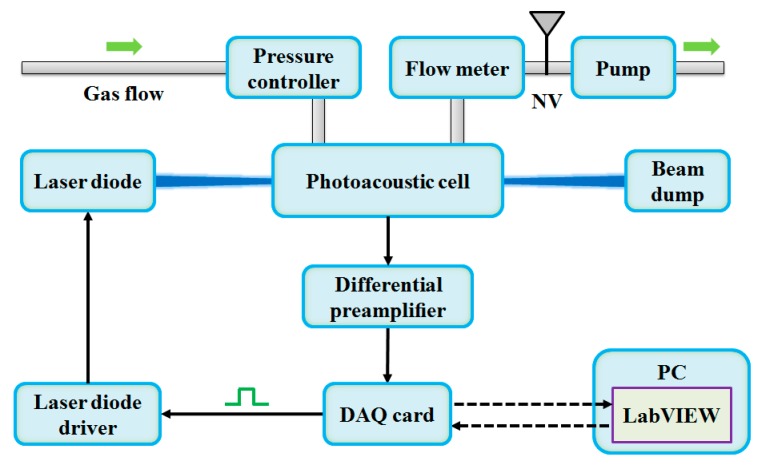
NO_2_ PAS sensor system. DAQ, data acquisition; PC, personal computer.

A compact PAS sensor system was developed for NO_2_ detection, as shown in Figure 4. A LabVIEW controlled data acquisition (DAQ) card (National Instrument USB-6361) generated a square signal with a duty cycle of 50% and a frequency of *f_0_* (the fundamental resonance frequency of the PAC) to drive the LD. The signals from two microphones were first processed by the differential preamplifier and then fed into the DAQ. A LabVIEW-based lock-in amplifier (LIA) demodulated the PAS signals in the 1-*f* mode. The filter slope and the time constant of the LIA were set to 12 dB/oct and 1 s, respectively, corresponding to a detection bandwidth of 0.25 Hz. All data were recorded and processed by a LabVIEW software program in a personal computer (PC). The pressure in the PAC was controlled and maintained by a mini diaphragm pump (KNF Technology N813.5ANE) and a pressure controller (MKS Instruments 649B13TS1M22M). A needle valve and a mass flow meter (Alicat Scientific M-2SLPM-D/5M) were used to adjust and monitor the gas flow rate, respectively. A 1 ppm NO_2_/air standard gas was diluted with zero air gas to produce NO_2_/air mixtures with different NO_2_ concentrations by means of a gas dilution system (Environics 4000).

## 3. Optimization and Evaluation of the NO_2_ Sensor

### 3.1. Parameters Optimization

#### 3.1.1. Laser Power Dependence of Photoacoustic Signal and Analysis of Saturation Effect

The laser power is important for a photoacoustic gas sensor. Generally, the amplitude *S* of the photoacoustic signal can be expressed as [24]:

(3) $$S=C_{cell}\cdot \alpha \cdot P$$

where *C_cell_* is the photoacoustic cell constant, α is the molecular absorption coefficient, and *P* is the laser power. At high laser power, Equation (3) may no longer be applicable because saturation occurs. Taking a simplified two-level system as an example, the absorption of the laser power causes gas molecules to be transferred to the excited state, from where they return to the ground state by spontaneous emission and/or collisional relaxation. At low laser powers the molecules in their ground states can maintain this process and the ratio of excited molecules, *N_e_*, and the total molecules number densities, *N*, can be described as [25]:

(4) $$\frac{N_{e}}{N}=\frac{\sigma I/h\nu }{2\left(\sigma I/h\nu \right)+k_{sp}+k_{r}}$$

where *σ* is the absorption cross section of the excited molecular transition, *I* is the laser intensity, *hν* represents the energy of the absorbed photons between the excited state and the ground state, *k_sp_* and *k_r_* are the rate constants of spontaneous emission and the effect of all relaxation processes, respectively. However, when the laser power is high enough, the following condition will occur:

(5) $$2\left(\sigma I/h\nu \right)\gg k_{sp}+k_{r}$$

At this level of irradiance, Equation (4) remains approximately constant at:

(6) $$\frac{N_{e}}{N}=\frac{1}{2}$$

**Figure 5:**
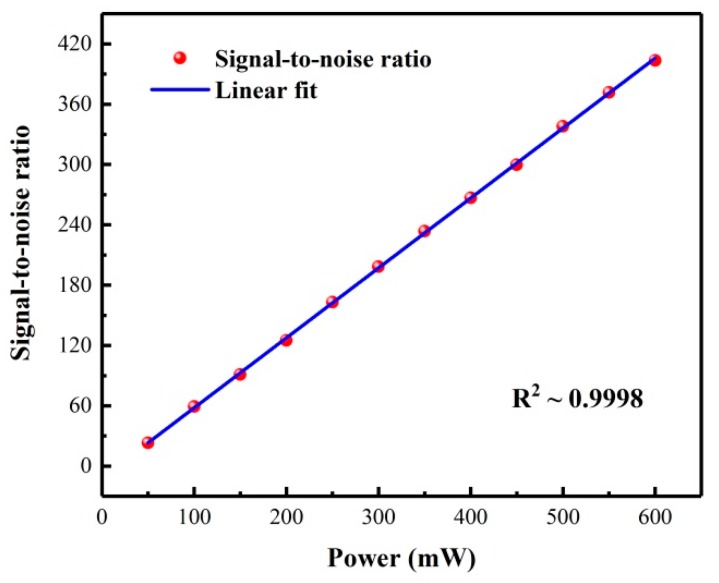
NO_2_ PAS signal-to-noise ratio as a function of the laser average power.

Hence, no more molecules are available to become excited to higher energy levels when the laser power increases further, which means that the transition is saturated. In order to avoid the nonlinear response of the sensor due to the saturation effect, a certified 100 ppb NO_2_/air gas mixture was fed into the PADM to check the saturation level. A large gas flow rate of 650 sccm was chosen for the reported sensor, which makes more unexcited gas molecules enter the PAC; they may become excited to higher energy levels, resulting in an enhanced photoacoustic signal and suppressed saturation effect [21]. Moreover, a large gas flow rate can reduce the gas exchange time, thus improving the response time. However, the noise of the photoacoustic sensor system may increase sharply when the flow rate increases. In our photoacoustic sensor, the designed compact low-noise photoacoustic detection module was employed to suppress the gas flow noise greatly as described in Section 2.2. The pressure in the PAC was maintained at atmospheric pressure. A laser power meter (Ophir Optronics Solutions, 3A-ROHS) was placed behind the PAC instead of the beam dump mentioned above to measure the modulated laser power. The measured SNRs with different laser powers are shown in Figure 5. The obtained R^2^ of 0.9998 by a linear fitting routine proves the linear response of the SNR to laser power and confirms that the reported sensor had not reached the saturation condition. Therefore, further experiments were carried out with a maximum laser average power of 600 mW to obtain the optimum detection sensitivity.

#### 3.1.2. Relationship between Photoacoustic Signal and Pressure

**Figure 6:**
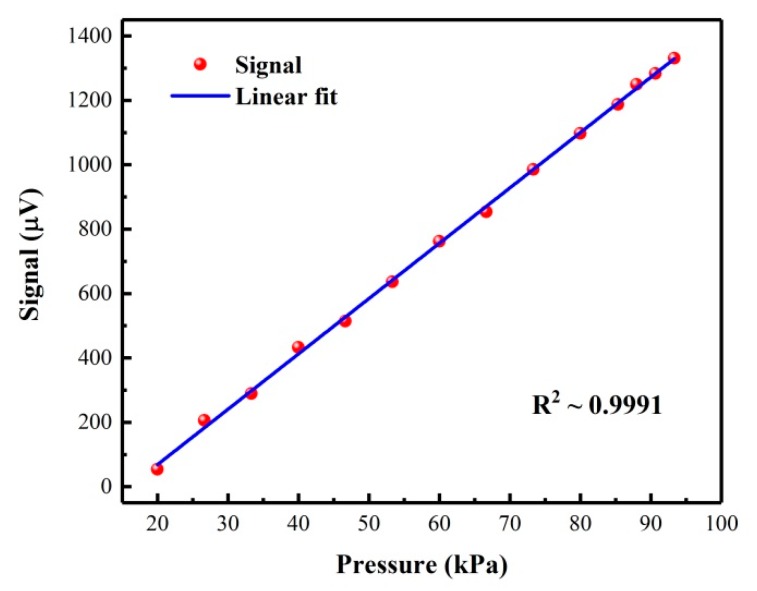
NO_2_ photoacoustic signal as a function of the pressure.

In the PAS technique, the photoacoustic signal is pressure-dependent, since the pressure can affect the excited acoustic resonance of the PAC and the vibration-translation relaxation (V-T relaxation) rate of the target gas [14,26]. In order to obtain optimum sensor performance, the operating pressure of the sensor must be optimized. A certified 100 ppb NO_2_/air gas mixture was used to select the optimum pressure. Figure 6 shows the relationship between the NO_2_ photoacoustic signal and different pressures, which indicates a linear increase of the NO_2_ photoacoustic signal (R^2^ ∼ 0.9991) with increasing gas pressure. We selected the atmospheric pressure as the operating pressure of the sensor, which can not only obtain the highest NO_2_ photoacoustic signal, but also remove the pressure controller to make the sensor more convenient and portable.

### 3.2. Performance Evaluation

**Figure 7:**
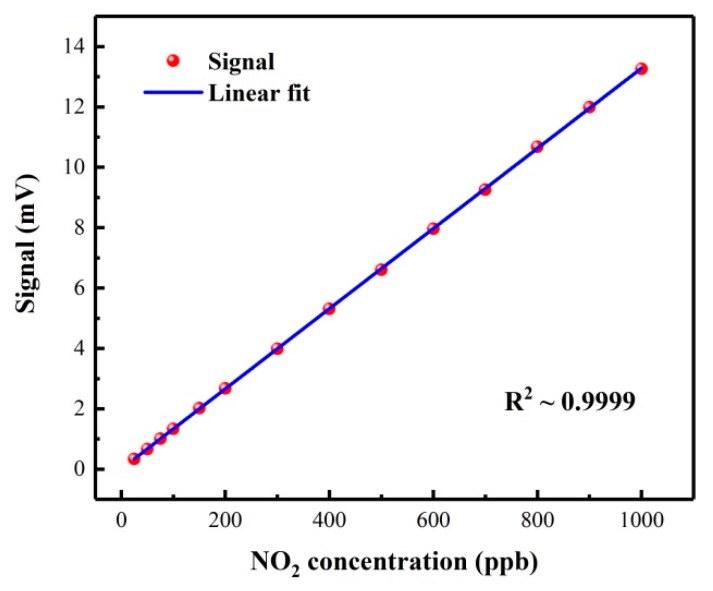
Response linearity of the NO_2_ photoacoustic sensor at different NO_2_ concentrations.

In general, the concentration of water vapor in ambient air is 0.5%–4%. In order to study the influence of water vapor on the photoacoustic signal, a 100 ppb NO_2_/air gas mixture was humidified with water vapor concentrations ranging from 0.5% to 4%. The results showed that the signal amplitude remained basically unchanged in this range, so the influence of water vapor was not considered when the reported NO_2_ sensor was applied to environmental monitoring. To evaluate the performance of the reported NO_2_ sensor, different concentrations of the NO_2_/air gas mixture were fed into the PADM while the sensor was operated with the optimum parameters mentioned above. The mean signal amplitudes were recorded at different NO_2_ concentration levels, as shown in Figure 7. A R^2^ value for linear fitting of 0.9999 confirmed the linearity response of the sensor to NO_2_ concentration levels. For a 50 ppb NO_2_/air gas mixture, a signal amplitude of 667.5 µV was obtained. Based on a noise level (3σ) of 3.3 µV, a SNR of 202.3 was calculated, which corresponds to a detection limit (3σ) of 250 ppt (part per trillion by volume) with a 1-s averaging time. The specific detectivity was determined to be 3.173 × 10^−9^ W cm^-1^ Hz^−1/2^.

## 4. Outdoor Continuous On-line Monitoring of Environmental NO_2_

**Figure 8:**
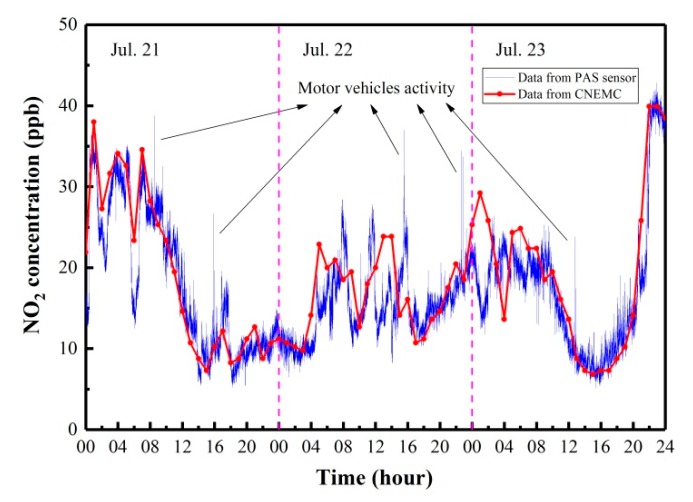
Continuous three-day monitoring of environmental NO_2_ concentrations measured in July 2019 on the Shanxi University campus, China (blue) and corresponding data available from a nearby station of the China National Environmental Monitoring Center (red).

The NO_2_ sensor was placed on the roof of the Yifu Building in Shanxi University, China, and a 72 h continuous on-line monitoring of environmental NO_2_ was carried out from 21–23 July 2019. The sensor was located inside a chamber that was made from lightweight polymethyl methacrylate. To reduce the heat transfer between the sensor and environment, the chamber was covered with thermal insulation material (Styrofoam). The air was continuously sampled into the sensor by a mini diaphragm pump. A 3-μm micropore hydrophobic polytetrafluoroethylene PTFE filter membrane was installed at the air inlet as an air filter to remove dust and soot particles. The three-day continuous measurement results of NO_2_ concentrations with a 1-s acquisition time are shown in Figure 8 (blue). A China National Environmental Monitoring Center (CNEMC), which used a chemiluminescence method for NO_2_ detection, was found ~3 km from our sensor system. For comparison, the NO_2_ concentration data released by the CNEMC website [27] with a very slow updating rate of 1 data point/h are also shown in Figure 8 (red). The variation in environmental NO_2_ concentration trends as measured by the PAS-based NO_2_ sensor is in excellent agreement with Figure 8 (red). Short duration oscillations can be clearly observed by our sensor due to the influence of wind and automobile activity, but these behaviors cannot be observed in Figure 8 (red) since all data within 1-h were averaged. In particular, several sharp peaks appeared at the time noted in Figure 8 (blue), which were related to motor vehicle activity near the sensor. At some specific moments, such as 5:00 a.m. (Beijing Time) on 22 July, 1:00 p.m. on 22 July, 2:00 p.m. on 22 July, and 1:00 a.m. on 23 July, the NO_2_ concentration values published by the CNEMC were higher than those obtained by our reported sensor. On the contrary, the concentration values published by the CNEMC were lower at 8:00 a.m. on 22 July and on 4:00 a.m. on 23 July. These small differences are mostly due to the distance between two sensor systems (~3 km) and local NO_2_ concentration variations.

## 5. Conclusions

A low-cost, compact, and highly sensitive NO_2_ sensor was developed for environmental monitoring. The PAS-based NO_2_ sensor employs a low-cost high-power LD to detect NO_2_ at 450 nm. A compact low-noise photoacoustic detection module was designed to operate together with the LD. The parameters of the sensor were studied in detail, and saturation-free NO_2_ detection was achieved. A 3σ detection limit of 250 ppt was obtained with a 1-s averaging time at atmospheric pressure. The corresponding specific detectivity is 3.173 × 10^−9^ W cm^−1^ Hz^−1/2^. The sensor was tested in a three-day continuous outdoor measurement run of environmental NO_2_ concentrations. The results are in excellent agreement with the data released by the CNEMC monitoring station, thus validating the performance of the NO_2_ photoacoustic sensor. Furthermore, the reported NO_2_ sensor could be employed in smart traffic lights to regulate traffic flow through cities and to reduce pollution hotspots.
